# Mechanisms of Adverse Local Tissue Reactions to Hip Implants

**DOI:** 10.3389/fbioe.2019.00176

**Published:** 2019-07-30

**Authors:** Felipe Eltit, Qiong Wang, Rizhi Wang

**Affiliations:** ^1^Department of Materials Engineering, University of British Columba, Vancouver, BC, Canada; ^2^School of Biomedical Engineering, University of British Columba, Vancouver, BC, Canada; ^3^Centre for Hip Health and Mobility, Vancouver, BC, Canada

**Keywords:** pseudotumors, corrosion, mitochondrial stress, tribocorrosion, fretting corrosion, metal hypersensitivity, total hip implants, adverse local tissue reactions

## Abstract

Adverse Local Tissue Reactions (ALTRs) are one of the main causes of hip implant failures. Although the metal release from the implants is considered as a main etiology, the mechanisms, and the roles of the released products are topics of ongoing research. The alloys used in the hip implants are considered biocompatible and show negligible corrosion in the body environment under static conditions. However, modularity and its associated mechanically assisted corrosion have been shown to release metal species into the body fluids. ALTRs associated with metal release have been observed in hip implants with metal-on-metal articulation initially, and later with metal-on-polyethylene articulation, the most commonly used design in current hip replacement. The etiological factors in ALTRs have been the topics of many studies. One commonly accepted theory is that the interactions between the metal species and body proteins and cells generate a delayed type IV hypersensitivity reaction leading to ALTRs. However, lymphocyte reactions are not always observed in ALTRS, and the molecular mechanisms have not been clearly demonstrated. A more accepted mechanism is that cell damage generated by metal ions may trigger the secretion of cytokines leading to the inflammatory reactions observed in ALTRs. In this inflammatory environment, some patients would develop hypersensitivity that is associated with poor outcomes. Concerns over ALTRS have brought significant impact to both the clinical selection and development of hip implants. This review is focused on the mechanisms of ALTRs, specifically, the metal release process and the roles of the metal species released in the etiology and pathogenesis of the disease. Hopefully, our presentation and discussion of this biological process from a material perspective could improve our current understanding on the ALTRs and provide useful guidance in developing preventive solutions.

## Introduction

Over one million people receive hip replacements every year to relieve pain and restore hip functions from osteoarthritis, a degenerative disease that affects most people in their seventh decade of life (RIPO, 2014; The Canadian Joint Replacement Registry, 2015). Although hip arthroplasty is generally a successful procedure, adverse local tissue reactions (ALTRs) can develop to the materials used in hip implants, affecting patients' health and decreasing their quality of life. ALTRs affect at least 10% of patients with Metal-on-Metal (MoM) hip implants as well as a lower but significant number of patients with Metal-on-Polyethylene (MoP) hip implants, the most commonly used system in total hip replacements (Matharu et al., 2016a).

The main symptoms of ALTRs are pain and swelling. They can generate extensive destruction to the soft tissues of the hip, challenging the prognosis of further clinical solutions (Williams et al., 2011; Almousa et al., 2013). The exact etiology of ALTRs is not clear. But their exclusive development after hip replacement suggests a link with the metal components of the hip implants (Cooper et al., 2013). It has been widely accepted that metal release can affect the periprosthetic tissues, leading to development of ALTRs. In order to guide clinical selection of orthopedic implants and facilitate the development of new implant technologies, there is a need to critically review current progress and clarify the mechanisms responsible for the development of ALTRs. In the following sections, we will start with corrosion and wear mechanisms from a material perspective, and then discuss the biological and immunological processes that led to the development of ALTRs.

## Materials and Designs in Hip Replacements

**Figure 1:**
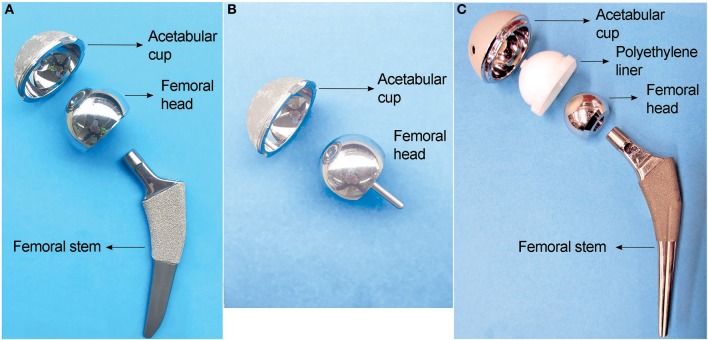
Three main types of hip implants. **(A)** Large head metal-on-metal (MoM) total hip implant. **(B)** MoM hip resurfacing. **(C)** Metal-on-polyethylene (MoP) total hip implant.

Metals have been commonly used in hip implants because of their excellent mechanical properties. Since the introduction of the highly biocompatible titanium alloys in 1970s (Albrektsson et al., 1981; Brånemark, 1983), a typical total hip replacement (THR) system is generally composed of a titanium femoral stem, a CoCrMo femoral head articulating with a polyethylene liner, supported by a titanium acetabular shell (Figure 1C). However, polyethylene wear debris generated in articulation against CoCrMo femoral head trigger osteolytic lesions, inducing inflammation and bone resorption (Ormsby et al., 2019). To minimize the wear of polyethylene in articulation, second-generation MoM articulation was developed in the 1990s (Figure 1A). The CoCrMo alloy used in articulating surfaces of MoM implants is composed of 60–65% cobalt, 27–30% chromium, 5–7% molybdenum, and 2–5% other trace elements as manganese, silicon, iron, nickel, and carbon (Schmidt et al., 1996). Through advanced technologies, it was possible to obtain smoother surfaces which theoretically would reduce wear and friction (Dowson, 2006). The assumption of lower wear was later demonstrated “*in vitro”* (Anissian et al., 1999; Clarke et al., 2000). Positive clinical outcomes were observed at that time (van der Bracht et al., 2011). Cr and Co ions levels in most of the patients' serum were considered acceptable, and there was no higher risk of cancer in patients with MoM compared to the general population (Mathiesen et al., 1995; Keegan et al., 2008; Makela et al., 2012; Lalmohamed et al., 2013). Due to these observations, MoM implants were considered safe to humans. The FDA approved the MoM systems for clinical use through the 510 K or “substantial equivalence” to previously cleared devices (Ardaugh et al., 2013).

To avoid dislocation, a common clinical complication, large femoral heads were introduced in the second-generation MoM hip implants in order to increase range of motion and joint stability (Kostensalo et al., 2012; Dargel et al., 2014). Tribological analyses demonstrated that there was no significant increase in friction between larger contact surfaces if the clearance between the surfaces was decreased (Rieker et al., 2005; Dowson, 2006). The possibility of having a metal femoral head, articulating directly over the metal acetabular cup, prompts the development of hip resurfacing (Figure 1B), a surgical procedure in MoM hip replacements that involves minimal bone removal on the femoral head. This design was especially effective for young and active patients, with good outcomes after 5 years follow-up, and a lower hospital stay (2.3 days) compared with total hip replacement (4.1 days) (Ward et al., 2011; Jameson et al., 2012; RIPO, 2014). Due to those advantages, 35% of THA performed between 1998 and 2008 in the US and Europe were MoM implants (Meier, 2010; Liao et al., 2013). Unfortunately, adverse reactions due to metals release started to be reported in 10–30% of the patients (Langton et al., 2011b; Smith et al., 2012; Almousa et al., 2013). They were clinically characterized by pain, rash, and the development of a local benign fibroma described as pseudotumor (Pandit et al., 2008; Mahendra et al., 2009). Subsequently most manufacturers have halted the production of MoM implants (Cohen, 2011).

Another technological improvement to hip prostheses was the development of modularity of the femoral components (Figure 1). The modularity allows surgeons to select the components independently to best fit patient's anatomy. It reduces the inventory in hospitals and manufacturers, and simplifies revisions procedure if needed (Srinivasan et al., 2012). However, the modular junctions between components experience corrosion due to fretting, which can be a source of metal particles or ions release responsible for the development of ALTRs (Fricker and Shivanatil, 1990; Brown et al., 1995; Kawalec et al., 1995; Kop et al., 2012; Wang et al., 2016).

## Mechanisms of Implant Degradation

**Figure 2:**
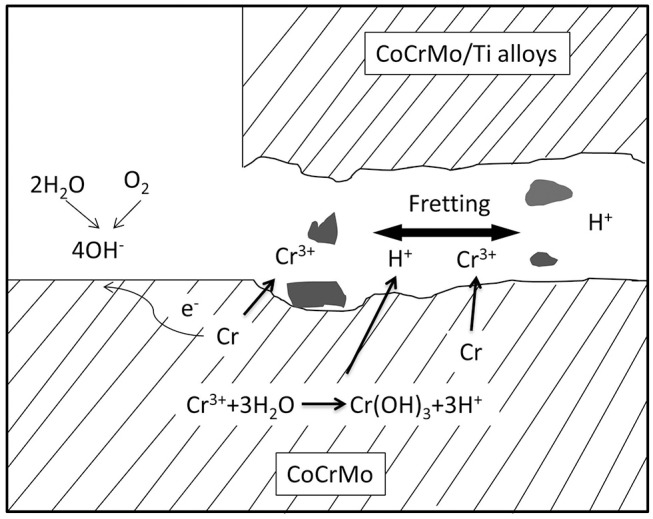
Illustration of fretting corrosion between two metallic components. Fretting corrosion is complexed by crevice corrosion that results in oxygen depletion and reduced pH in the crevice where fretting happens.

The metal alloys used in hip replacements are considered to be corrosion resistant, mostly due to the protection provided by a dense 2–4 nm-thick passive oxide layer formed on their surfaces. Laboratory studies showed that Ti6Al4V and CoCrMo alloys would not experience significant corrosion when they contact each other under simulated physiological conditions in static environment (Lucas et al., 1981; Griffin et al., 1983). However, the presence of corrosion products at the modular junction of the retrieved implants, and elevated metal ions in the serum of patients have been widely reported since 1990s (Collier et al., 1990, 1992; Mathiesen et al., 1991). This corrosion process is attributed to the disruption of the passive film caused by mechanical wear (Brown et al., 1995; Jacobs et al., 1998). There are two possible locations where this mechanically assisted corrosion is likely to happen: (i) the articulating surfaces, where the friction between the moving femoral head and acetabular components generates wear on the surfaces and accelerates corrosion. This material degradation process is often referred as tribocorrosion. (ii) the modular junction of the implants, in which the cyclic load generates micromotion (fretting) at the interface of the components, causing destruction of the passive layers on both surfaces and subsequent corrosion of the underlying metals. This fretting corrosion at the modular junction is enhanced by the presence of crevice corrosion, which is characteristic in confined space of the tapper junctions of modular implants (Figure 2). The reactions that occurs inside the crevice are initiated by the mechanical removal of the passive layer (fretting corrosion). Subsequently, the surfaces re-passivate, consuming oxygen in the reaction. After each cycle and due to the confined space, oxygen is depleted, which further retard the formation of a new passive layer and increase corrosion rate (Jacobs et al., 1998).

Mechanically assisted corrosion of metal alloys in hip implants releases solid particles as well as metal ions into the peri-implant environment. It is generally accepted that those released metal species would trigger adverse tissue reactions which ultimately require revision surgery. It is thus necessary to first discuss our current understanding on the *in vivo* metal release process and the nature of the degradation products in terms of morphology, composition, crystallinity, and concentration.

Another important factor regarding the corrosion of hip implants is the presence of biological products interacting with the implants' surfaces. The effects of biological elements on the degradation of materials is referred as biocorrosion (Cadosch et al., 2009). The articulating surfaces and modular junctions of the hip implants are surrounded by the synovial fluid, which constitutes a relatively mild environment of buffered solution at around pH of 7.4 with temperature of 37°C (Hanawa, 2004). However, there are some specific bio-corrosive elements such as ions, proteins, proteoglycans, biologically induced pH changes, and oxidizers, that may affect its physicochemical behavior (Talha et al., 2019). The presence of proteins in the physiological environment may affect the corrosion of metallic materials by changing the mechanics and kinetics of the corrosion reactions on the surface (Goldberg and Gilbert, 1997; Hallab et al., 2003; Okazaki and Gotoh, 2005; Valero Vidal et al., 2010; Igual Munoz et al., 2015). A decrease in the pH is usually a consequence of inflammation or hypoxia, produced by the enhanced glycolytic pathways and subsequent increase in the production of lactate or pyruvate. This decrease in pH could theoretically enhance corrosion of the implants alloys; Additionally, active oxygen species released by immunological cells such as macrophages when responding to a foreign body or during inflammation are potential oxidizers that could enhance the corrosion process (Hanawa, 2004; Gilbert et al., 2015; Liu and Gilbert, 2017).

### Particles Release From Tribocorrosion at the Articulating Surfaces

Tribocorrosion at the articulating surfaces has been studied both through retrieval analyses and “*in vitro”* laboratory wear tests by using hip simulator in simulated body fluid (Table 1). Material release from implants depends on the design and material composition. In general, the size of particles generated is on average 300 nm for polyethylene, 30 nm for metal and 9 nm for ceramic (Tipper et al., 2001). Although the volumetric wear in MoM articulations is about one tenth of the polyethylene wear observed in zirconia on polyethylene or MoP implants, the nature of the particles released presents a higher risk for the development of adverse local tissue reactions (Goldsmith et al., 2000). Analyses on articulating surfaces of MoM implants have proposed that wear (tribocorrosion) on CoCrMo alloys may be responsible for hip implant failure (Campbell et al., 2006; Matthies et al., 2011; Al-Hajjar et al., 2013).

**Table 1:** Summary of reported corrosion debris from CoCrMo hip implants.

**Locations**	**Corrosion debris and representative reports**
Head-neck junction	**Inside:** crystalline metal oxides containing Cr, Mo with depleted Co (Urban et al., 1994, 1997)
	**At the opening:** amorphous chromium (III) phosphate hydrate (Urban et al., 1994, 1997)
Peri-implant tissues	**MoM implants:** oxide particles containing Cr^3+^ (Catelas et al., 2004, 2006; Goode et al., 2012);
	Nano-sized metallic particles (Doorn et al., 1998; Catelas et al., 2004, 2006; Goode et al., 2012; Topolovec et al., 2013; Bitounis et al., 2016);
	Chromium (III) phosphate (Huber et al., 2009; Hart et al., 2010, 2012).
	**MoP implants:** oxide particles containing Cr with low Co and sub-micron metallic particles (Shahgaldi et al., 1995; Urban et al., 2000; Topolovec et al., 2013); chromium phosphate (Clarke et al., 2003; Illgen et al., 2008).

**Figure 3:**
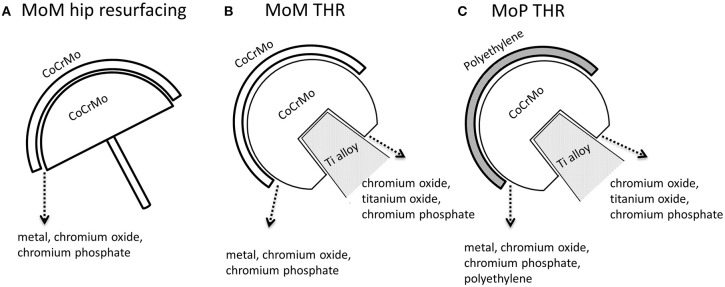
Types of particles released from the femoral head-cup articulation and head-neck junction of CoCrMo based hip implants. **(A)** MoM hip resurfacing. **(B)** MoM total hip implant. **(C)** MoP total hip implant. In addition to polyethylene (PE) and titanium oxide particles, metal particles are particles with similar chemical composition to the alloy CoCrMo; chromium oxide is a type of particles containing Cr, O with low or depleted Co; chromium phosphate particles contain Cr, O, P, Ca with low or depleted Co in EDS spectrum.

Hip simulation studies showed that CoCrMo metallic particles and chromium oxide nanoparticles are the main particles released as a consequence of tribocorrosion in Metal-on-Metal articulation (Figures 3A,B; Firkins et al., 2001; Catelas et al., 2003). The generation of wear particles is also affected by loading conditions. Under edge-loading, CoCrMo nanoparticles generated were relatively larger; while under normal loading, most particles generated are smaller chromium oxide nanoparticles (Kovochich et al., 2018).

The particles released “*in vivo”* from the articulating surfaces by tribocorrosion are not retained to the surface of implants. Since there are no modular junctions in MoM hip resurfacing system (Figure 1B), tribocorrosion at the articulating surface is the sole source of metal release. Analysis of corrosion products in synovial fluid and tissues from MoM hip resurfacing offer insights on particles associated with tribocorrosion at the metal bearing surfaces. By use of *SEM-ED*X analysis, three types of particles have been observed in tissues surrounding MoM hip resurfacing: chromium oxide, CoCrMo metal, and chromium phosphate particles (Doorn et al., 1998; Hart et al., 2010; Goode et al., 2012). The oxidation state of these particles is Cr (III) (Hart et al., 2010; Goode et al., 2012). Compared to laboratory studies, “*in vivo”* studies revealed another type of particle—chromium phosphate particles. Similar chromium phosphate compound was also commonly observed in the tissues from MoM THR and MoP THR. The formation of this type particles is speculated to be the reaction product of Cr ions with the body fluid containing phosphate ions.

### Particles Released From Fretting Corrosion at the Modular Junctions

Fretting corrosion has been demonstrated to affect modular interfaces of implants composed of similar or different alloys, as observed in systematic analysis of retrieved implants (Table 1) (Gilbert et al., 1993). But limited experimental studies have analyzed variables such as materials, electrolytes and load to explain the mechanisms associated with these variables (Swaminathan and Gilbert, 2012).

An early “*ex-vivo”* study led by Jacobs et al. (1995), analyzed the morphology and the chemical compositions of corrosion products at the modular interfaces of retrieved femoral heads. The study concluded that the composition of corrosion compounds varies along the topographical locations. Chromium oxides and chlorides existed inside the tapper junction, in the contact area between head and neck, but chromium orthophosphate was found mainly at the rim of the head bore and, on the neck surface distal to the head-neck junction (Jacobs et al., 1995). Another study from the same group in 1997 described that at the contact area, the products corresponded to mixed crystalline oxides of Cr, Mo, and Ti, while in the open crevice, the products were mostly amorphous hydrated chromium orthophosphate. Interestingly, minimal cobalt was found in the corrosion particles on the implant surfaces (Mathiesen et al., 1991; Urban et al., 1997). These observations were later confirmed by other researchers (Huber et al., 2009; Chana et al., 2012; Baleani et al., 2017). The Cr-rich fretting corrosion products with low or depleted Co indicated that Co was released in the form of soluble species.

**Figure 4:**
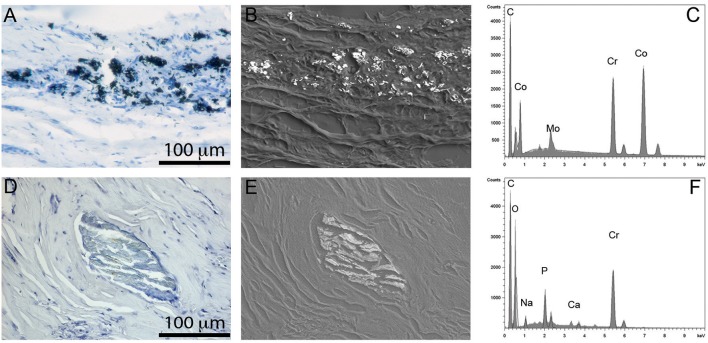
Particles observed in periprosthetic tissues. **(A–C)** CoCrMo metal particles of sub-micron size group in leukocyte infiltrated areas **(A)**; they show a high contrast in back-scattered electron image due to their high density **(B)**; the EDS spectrum shows peaks of Co, Cr, and Mo **(C)**. **(D–F)** chromium phosphate particles, tens to hundreds of microns in size usually surrounded by a collagenous capsule **(D)**, showing low density in back scattered electron image **(E)**, and peaks in Cr, O, and P in EDS spectrum **(F)**.

Since MoM THR consists of articulating surfaces as well as the head-neck junction, the presence of solid particles in periprosthetic tissues can be associated with both tribocorrosion at the articulating surfaces and fretting corrosion at head-neck junction (Figures 3, 4). In tissues and synovial fluid surrounding MoM THRs, oxide particles containing Cr as well metallic nano-particles were observed (Doorn et al., 1998; Catelas et al., 2004, 2006; Ward et al., 2010; Topolovec et al., 2013); corrosion particles rich in chromium and phosphate were also observed (Huber et al., 2009; Xia et al., 2017).

In MoP hip implants, most studies assumed negligible wear on metal head because of the relatively low wear resistance of polyethylene. It was commonly accepted that particles observed in tissues surrounding MoP hip implants were mainly associated with fretting corrosion at the head-neck interface. Chromium and phosphate rich particles were the primary debris detected in the tissues surrounding MoP hip implants (Cooper et al., 2013). However, we recently observed the presence of extensive wear on the articular surfaces of CoCrMo femoral heads in MoP hip implants, which demonstrated that tribocorrosion could also result in significant metal release in MoP hip implants.

### Metal Ions Release From Metal Implants

The presence of elevated metal ions in serum and synovial fluid have been associated with the development of ALTRs (Amstutz and Le Duff, 2017). Monitoring Co and Cr levels in serum or whole blood is the current clinical predictor of hip implants malfunction and provides alert to the development of ALTRs. The metal release is directly associated with mechanically assisted corrosion, such as tribocorrosion and fretting. Mechanically assisted corrosion not only releases solid particles, but also has a tremendous impact on metal ions release. Especially, the metal ions are released by multiple mechanisms. The wear disrupts the passive layer on the implant surface, re-passivates and releases metal ions. The nano- and micron-sized particles generated by wear may further release ions when exposed to body fluids due to their large surface areas. Cell reactions to the particles generate an oxidizing environment. It has been observed that phagocyted metal particles by tissue macrophages are processed in lysosomes, which are known to be highly oxidative structures, capable of increasing the release of metal ions from the phagocyted particles (Park, 2003; Billi and Campbell, 2010).

Cobalt (Co) is preferentially released as Co^2+^ during the formation of the passive film (mainly chromium oxide) on the implant surfaces (Li et al., 1999; Milošev and Strehblow, 2003; Hanawa, 2004). Cobalt salts are highly soluble, and do not tend to precipitate in solution (Foote, 1923). A immersion study of CoCrMo alloy in phosphate buffered solution (PBS) demonstrated that Co^2+^ was the most abundant ion released from the alloy with a ratio Co:Cr:Mo = 13:2:1 (Hedberg and Odnevall Wallinder, 2014). Interestingly in the same study it was observed that cobalt release rate was higher in PBS enriched with albumin, which suggests a cobalt-protein interaction that affects the electrochemical reaction of Co. This enhanced effect was not observed in Cr or Mo.

Chromium (Cr) is the major elemental component in the passive layer formed on the CoCrMo surfaces. It is released from the implants as Cr^3+^ at extremely low rate in static immersion tests compared with Co^2+^ and Mo (Metikos-Huković et al., 2006). However, after long-term implantation of CoCrMo alloys, elevated levels of Cr^3+^ ions were observed in blood and urine of patients with metal hip replacements (Lhotka et al., 2003). This release of chromium is believed to be associated with disruption of passive film due to mechanical wear. Chromium compounds are believed to be formed rapidly after ion release, by reaction with phosphate in the synovial fluid and formation of highly insoluble precipitates (Ness et al., 1952; Rai et al., 1987).

Molybdenum (Mo) is rarely studied in the research associated to ALTRs. However, the lack of studies in synovial fluid does not exclude the possible adverse effects of Mo on the periprosthetic tissues. Molybdenum species are stable in passivity at low pH, but a pH higher than 7.0 is thermodynamically favorable for Mo dissolution (Martin et al., 2013). Moreover, molybdenum oxides formed on the metal surfaces exposed to air, disappeared quickly after immersion in 0.15 M NaCl (Li et al., 1999). Although traditionally it was not considered an important factor, its interaction with proteins recently revealed an important role in the dynamics of molybdenum release. A study using Electrochemical Quartz Crystal Microbalance reported that protein deposition occurs preferentially on Mo surfaces (Martin et al., 2013). Furthermore, by improving protocol for metal ion measurement with use of extended centrifugation and ultrafiltration, researchers revealed an accelerated release of molybdenum ion from CoCrMo particles in the presence of bovine serum albumin compared to PBS alone (Simoes et al., 2016).

Co and Cr concentrations in blood have been often monitored to evaluate the performance of hip implants. Comparison studies revealed that Co and Cr ions in the blood of patients with ALTRs were significantly higher than the control group without ALTRs (Kwon et al., 2011; McGrory et al., 2017). Concerns over metal over release in patients with MoM hip replacements were raised shortly after the introduction of the second generation MoM implants (Savarino et al., 2002). In 2015, UK Medicines and Healthcare Regulatory Agency (MHRA) issued a blood cobalt and chromium guidance value of 7 μg/L to identify MoM hip implant patients who may need further surveillance on excessive implant wear (archived in 2017) (Medicines Healthcare products Regulatory Agency, 2015). Similarly, the American Association of Hip and Knee Surgeons, the American Academy of Orthopedic Surgeons, and the Hip Society recommends a systematic evaluation of patients with dual modular neck systems, to include the analysis of serum ions levels to optimize the management of these patients (Kwon et al., 2014). Although cobalt and chromium in serum have been proposed as a monitoring tool for the performance of CoCrMo hip implants, it has been demonstrated that their analysis only has a modest sensitivity and specificity (60%) in identifying patients with ALTRs in MoM hip implants (Malek et al., 2012; Matharu et al., 2016b).

Despite the challenges to define thresholds of Co or Cr ion concentration in serum as predictors of ALTRs, the relative Cr/Co in serum provide insights on the releasing mechanisms. Most reports revealed a higher Cr/Co in serum from patients with MoM hip resurfacing (without head-neck junction) than patients with MoM total hip replacement. One study revealed 1.73 and 2.15 of mean Cr/Co in serum from total 160 cases of two types of hip resurfacings (Langton et al., 2009), much higher than 0.67 of mean Cr/Co from 17 patients with MoM total hip replacement (Eltit et al., 2017). This is consistent with the direct comparison showing the higher Cr/Co in serum from hip resurfacings than MoM total hip replacements (Garbuz et al., 2010). Another report also supported a much higher Cr/Co (0.91) in blood in hip resurfacing group than in total hip replacement group (0.53) (Matharu et al., 2017). These reports suggested that Cr in serum was preferably released from tribocorrosion at the bearing surface rather than fretting corrosion at the head-neck junction.

Compared to MoM hip implants, concentrations of Co and Cr ions in serum of patients with MoP total hip implants were far less studied. Metal ions in patients with well-functioning MoP total hip implants are usually analyzed as the control to compare with patients with MoM hip implants (Savarino et al., 2002; Cobb and Schmalzreid, 2006). To the best of our knowledge, there are no alerts regarding metal or serum ion levels in MoP systems, but a recent study proposed a threshold of 1 μg/L as cutoff for identification of ALTRs in patients with MoP hip implants (Fillingham et al., 2017). Our study revealed increased Co and Cr ions in serum of patients with ALTRs in MoP compared to ones without ALTRs (Eltit et al., 2017). These results indicated metal species released from CoCrMo implants were the stimuli responsible for developing ALTRs in patients with MoP total hip implants. Our latest results on metal ions measurement combined with wear analysis showed elevated Cr ion in serum with increasing roughness of CoCrMo femoral head in retrieved MoP hip implants. It indicated that Cr ions in serum were mainly attributed to the tribocorrosion at the articulating surface. In addition, we also showed that accelerated tribocorrosion also resulted in high level of Co ions in serum of patients with MoP hip implants.

Compared to metal ions in blood, far fewer studies had been reported on metal ions in synovial fluid. Hip implants are exposed to the joint fluid, a viscous fluid produced by the synovial membrane. It contains hydrophilic molecules such as hyaluronic acid that increase its viscosity and reduce friction. The concentration of Co and Cr ions in the synovial fluid of patients without implants is around 1 μg/L and 4 μg/L, respectively. These numbers could increase to a range between 50 and 100 μg/L in patients with well-functioning implants and could reach between 500 and 10,000 μg/L in patients with failed implants (Lass et al., 2014; Eltit et al., submitted). Compared to serum, it has been observed that the Co and Cr ions concentrations in synovial fluid have greater variations and are relatively higher (De Smet et al., 2008; Davda et al., 2011; Eltit et al., 2017).

## ALTRs

### Definition and Nomenclature

Adverse Local Tissue Reactions (ALTRs) have been histologically described as the growth of cystic or solid fibrotic masses originating in the synovial membrane of patients with hip implants (Doorn et al., 1996; Mahendra et al., 2009; Natu et al., 2012; Perino et al., 2014; Matharu et al., 2016a). ALTRs generate pain and discomfort, as well as pressure on vein and nerves, but some can also be asymptomatic (Maurer-Ertl et al., 2011; Konan et al., 2017).

Different terms have been used to describe lesions associated with hip replacements. The clinical name “Pseudotumor” was used in the 1980s to describe soft tissue growth around hip implants (Griffiths et al., 1987), it was thought to be a reaction to the acrylic cement of the femoral components but was later observed in non-cemented systems (Svensson and Mathiesen, 1988). The same term was reincorporated for the lesions found in second generation MoM implants in the study of Pandit et al. (2008). Pseudotumor refers to the Greek “pseudo” for false and the Latin “tumor” for increase in volume. The actual pathology observed surrounding the implants corresponds to a true volume increase, but since the term “tumor” has been associated with neoplasms, the prefix pseudo was added to reduce concerns over the malignancy of these inflammatory lesions. Pseudotumor then, corresponds to a clinical and imaging description of a benign volume growth associated with a hip replacement system. Aseptic Lymphocyte-dominated Vasculitis-Associated Lesion, was introduced by Willert et al. (2005). Although the acronym ALVAL was used for the first time by Campbell et al. (2010) who also introduced a method of quantifying the severity of the lesions based on the histological observations (ALVAL score), which is now been used in most of the studies to describe the severity of the lesions around hip implants (Campbell et al., 2010). The term ALVAL corresponds to the histological description of the lesions that are found in the clinically described “pseudotumors.” Adverse Reaction to Metal Debris (ARMD) was introduced by Langton et al. (2010). ARMD is used to define the pathology associated with MoM implants which suggests that the failure is due to the presence of wear debris (Langton et al., 2010), which was supported by an increased wear rate in failed implants (Langton et al., 2011a). However, it indicates that metal debris are the main cause of the adverse reactions, which is still under investigation. Adverse Local Tissue Reactions (ALTRs) is a term that includes a broad spectrum of pathological changes associated with the presence of hip implants. It was used in the 1970s and 1980s to describe the pathology in the tissues surrounding hip implants (Forest et al., 1985), and was reintroduced to describe the failure of the second-generation MoM hip prosthesis in a literature review by Carli et al. (2011). It is currently the most frequently used term to define the aseptic periprosthetic lesions (Carli et al., 2011).

### Histological Characteristics of ALTRs

**Figure 5:**
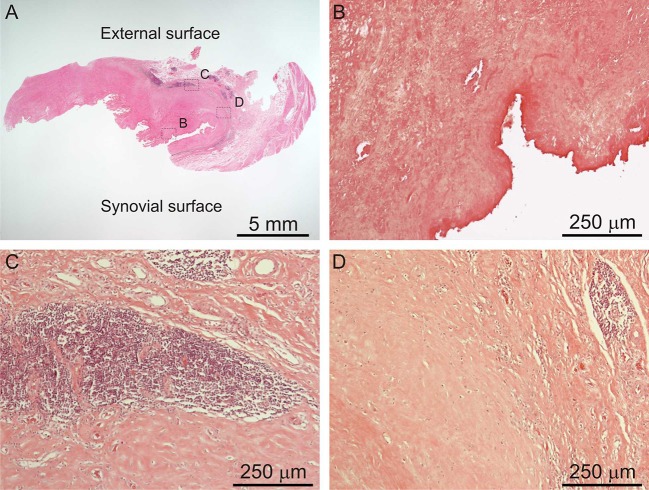
Histological images of ALTR. Low magnification of biopsied lesions **(A)**, characterized by synovial ulceration and sub superficial necrosis **(B)**, mononuclear cell infiltration **(C)**, and macrophages **(D)**.

ALTRs are described as solid masses or cystic cavities, in which the ulceration of the synovial surface with or without fibrin deposition, is accompanied by sub superficial necrosis, mononuclear cell infiltration and variable number of eosinophils and giant multinucleated cells, in a thickened synovial membrane composed of dense connective tissue (Figure 5; Mahendra et al., 2009; Natu et al., 2012; Perino et al., 2014).

**Figure 6:**
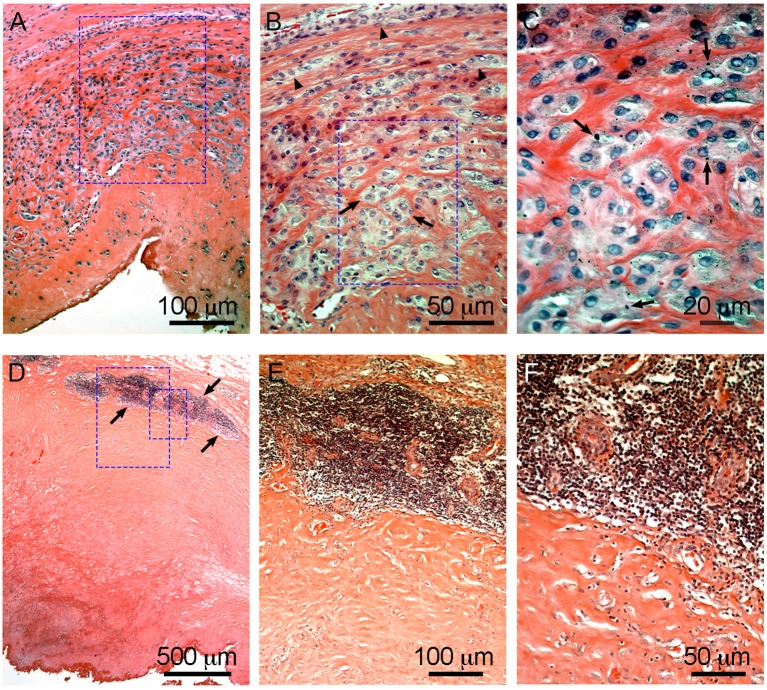
Characteristic macrophage-dominated ALTR **(A–C)** and ALTR with lymphocyte infiltration and hypersensitivity features **(D–F)**. Macrophage dominated lesions are associated with evidence of high wear, corrosion, and elevated Co and Cr in serum (53 and 48 μg/L, respectively, in the case shown). ALTRs with predominant lymphocyte infiltrate and hypersensitivity features usually are not associated with elevated wear or corrosion and may be present with lower Co and Cr in serum (6.2 μg/L and 1.2 μg/L in the case in **D–F**). Low extension of sub superficial necrosis is observed in macrophage-dominated lesion with macrophages that infiltrate the whole synovium **(A)**, forming clusters (arrows) or epithelioid cells (arrowheads) in **(B)**, and multiple micron/sub-micron particles (arrows) are observed in macrophages cytoplasm **(C)**. Hypersensitivity feature ALTRs usually present with large extension of sub superficial necrosis **(D)**, with perivascular lymphocyte infiltration that form large aggregates (over 1 mm) and may constitute tertiary lymphoid organs (arrows in **D,E**), with low (or none) number of particles observed in the tissues **(F)**.

It is accepted that, as in most foreign body reactions, the immune system plays a critical role in the development of ALTRs. This is supported by the histological observations of macrophages and T cells in the tissue, occasionally with giant multinucleated cells and eosinophils (Mahendra et al., 2009). Generally, lesions with a large number of macrophages presenting a low lymphocyte infiltration and highly lymphocyte-infiltrated lesions do not exhibit large number of macrophages (Figure 6) (Campbell et al., 2010; Ricciardi et al., 2016). The T-cell infiltration varies from the diffuse presence of T cells that has been found to have an equivalent number of CD4+ and CD8+, to the presence of large perivascular mononuclear aggregates composed of T (CD3+) and B (CD20+) cells, which has been defined as a characteristic of high-grade ALTRs (Natu et al., 2012). By use of immunohistochemistry, a high level of organization has been observed in the perivascular aggregates in which the B cells are located in the central zones (follicles) surrounded by T cells (Mittal et al., 2013). Because of this organization, and the molecular interactions between cells have been proposed corresponding to tertiary lymphoid organs, similar to those observed in peripheral organs with chronic inflammation (Natu et al., 2012; Neyt et al., 2012; Mittal et al., 2013). The phagocytic role of macrophages in ALTRs has also been reported, and the presence of metal particles inside the macrophage has been confirmed by optical microscopy (Figure 6C) or transmission electron microscopy (TEM) (Huber et al., 2009; Xia et al., 2011). There is an association between the amount of wear on implant surfaces and the number of macrophages in the tissues (Campbell et al., 2010). TEM observations of living macrophages within the tissues have shown that the particles were inside the phagosomes. But when the cells died, the membranes disintegrated, and the particles were released (Xia et al., 2011).

### Etiology of ALTRs: Role of the Immune System, Hypersensitivity Hypothesis

**Figure 7:**
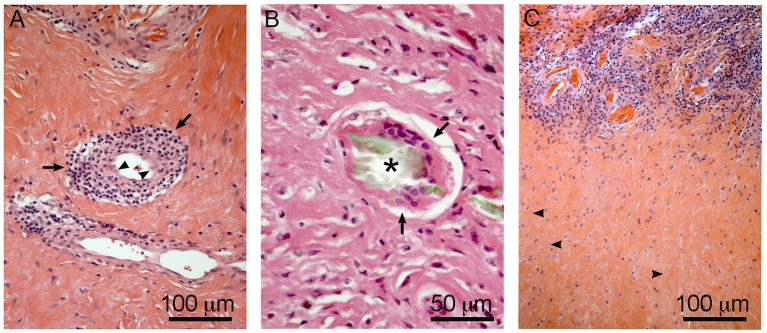
Hypersensitivity features in ALTRs. Perivascular T cell infiltration/vasculitis (arrows) observed in **(A)**, note the changes in the morphology of the endothelial cells to rounded or prismatic (arrowheads). Giant multinucleated cell (arrows) in **(B)**, frequently they are associated with large corrosion-product (Cr rich, no Co) particles (asterisk). T cell infiltration zones are usually surrounded by necrotic areas with low vascularity and evidence of cell degeneration (arrowheads) **(C)**.

The products of material degradation (both metal ions and solid particles) released from the implants as a consequence of corrosion or wear, have been accepted as the main etiological factor of ALTRs (Langton et al., 2011b). Nevertheless, studies have failed to correlate the amount of wear or corrosion in the implants with the severity of the lesions and clinical observations (Schmalzried, 2009; Campbell et al., 2010). These results, together with histological descriptions support the hypersensitivity theory that ALTRs correspond to a type IV or delayed hypersensitivity reaction triggered by metals (Huber et al., 2009; Mahendra et al., 2009; Thomas et al., 2009). Type IV hypersensitivity corresponds to the harmful effects of the cell-mediated immune response, in which the damage generated by the immune system is exaggerated in comparison to the damage that the etiological agent may produce (Abbas et al., 2015). Activated perivascular T-cells trigger cell death and secrete cytokines that induce macrophages to fuse forming giant multinucleated cells around the damaged area (granuloma), and also stimulate the proliferation of fibroblasts leading to tissue fibrosis (Kumar et al., 2012). All these features (T-cell infiltration, vasculitis, giant multinucleated cells, cell death, granuloma, and fibrosis) are found in ALTRs (Figure 7), providing strong support to this theory (Natu et al., 2012; Perino et al., 2014; Ricciardi et al., 2016).

The possible evidence of the interaction between metal species and T-cell is the decrease in the total number of circulating T lymphocytes observed in patients with MoM implants (Granchi et al., 2003; Catelas et al., 2015), and the CD8+ T-cell lymphopenia associated with high concentration of cobalt and chromium ions in plasma (Hart et al., 2009). It is thought that Co^2+^ interferes with antigen presentation, inhibiting T-cell expansion. However, this decrease in circulating T-cells could also be due to the local sequestration that occurs in periprosthetic tissues.

It is not clear how metals can induce hypersensitivity, but a molecular mechanism by which metals ions induce T-cell mediated hypersensitivity (type IV) has been recently described for beryllium (Be). The Be^2+^ ion conjugates with part of the HLA-peptide complex (HLA-DP2) and generates structural changes in the molecule. This modified-HLA molecule is recognized by the T-cell receptor, triggering the immune reaction responsible for severe lung destruction due to chronic inflammation in 1–15% of people exposed to beryllium (Clayton et al., 2014; Kugelberg, 2014; Fontenot et al., 2016). A similar pathway involving T-cell receptor activation may also be part of the mechanisms of immunogenicity triggered by the metals from the implants. Quite a few researchers have proposed that metal ions or nanoparticles can act as haptens or “incomplete antigens,” that combine with host proteins to generate a “neoantigen” (a new antigen that the immune system has not been exposed before) that activates T cell receptors (Hallab and Jacobs, 2009; Athanasou, 2016). Nevertheless, a study of lymphocyte activation in the presence of cobalt and chromium showed no differences between the lymphocytes obtained from patients with failed MoM implants and the control group, which suggests that metal hypersensitivity may not be the main mechanism in the development of ALTRs (Kwon et al., 2010). Clinical studies have also shown no association between metal allergy and implant failure (Thyssen et al., 2009). Further analyses demonstrate that the frequency of positive metal sensitivity test increases in patients who receive a hip replacement, and the increase is even higher in patients with failed implants; however, the test is not predictive for ALTRs (Granchi et al., 2012). Currently in most surgical centers all patients who are receiving hip implants are tested for metal allergy before surgery. And CoCrMo alloys are not used in patients with allergy. Nevertheless, a high rate of ALTRs is still observed in patients with MoM implants. These observations, together with the higher risk on patients with elevated Co^2+^ and Cr^3+^ ions in blood, suggest that a complex process involving multiple mechanisms such as cellular damage induced by metal ions, cell death, and immunogenicity of metal ions (Harinderjit et al., 2012).

### Etiology of ALTRs: Hypothesis of Cellular Hypoxic Stress

The roles of cobalt and chromium products in triggering the cellular phenomena observed in ALTRs such as inflammation, cell death and fibrosis, have been partially demonstrated “*in vitro.”* Studies carried out at the Rizzolli Orthopedic Institute had shown that high concentrations of Co^2+^ ions generated necrosis in human peripheral mononuclear cells, while lower concentrations induced apoptosis in the same cell population. On the other hand, Cr^3+^ ions at high concentrations induced apoptosis in the cells and no effects were observed in lower concentrations (Granchi et al., 1998a). Another study in macrophage-type cells (J774), showed that after 24 h of exposure to elevated cobalt and chromium concentrations the cell death was due to apoptosis. But after 48 h of exposure, the cells experienced necrosis (Huk et al., 2004). The same group, in a later report by Catelas et al. (2005) showed that chromium need higher ion concentrations than cobalt (250 vs. 8 ppm) to generate similar effects in J774 cells, suggesting that elevated concentration of these ions *"in vivo”* could be responsible of the cell death in the tissues (Catelas et al., 2005).

Cobalt and chromium particles also have toxic effects on human cells. Studies in keratinocytes (HaCaT cells), cultured with different concentration of nanoparticles of Cr_2_O_3_ showed the increased cell death due to apoptosis with the increased number of particles (Horie et al., 2013). It was later demonstrated that the cell death induced in a macrophage cell line (J774) by nanoparticles of chromium oxide corresponded to apoptosis (VanOs et al., 2014). A study using dermal fibroblast showed that nanoparticles of CoCrMo alloy produced a higher rate of cell death than a similar volume of micron-size particles; The authors then proposed that the higher surface area of the nano-sized particles generated a higher ion release (Papageorgiou et al., 2007). These effects of metal particles on cell viability are thought to be associated with release of metal ions that have direct effects on cells. It has been demonstrated that cobalt ions produce significantly higher cytotoxicity than cobalt particles in lung fibroblasts (Smith et al., 2014), and the generation of reactive oxygen species (ROS) in T-cells is induced by Co ions but not by nano- and micron sized particles (Chamaon et al., 2019).

The described difference in toxicity of cobalt and chromium may be attributed to their difference in solubility and the capability to penetrate the plasma membrane. It was explained previously that chromium tends to precipitate in the presence of phosphate ions, the common species present in human tissues, decreasing the concentration of “free ions” able to penetrate the cells and cause deleterious effects. Chromium usually exists in the form of Cr^3+^ in synovial tissues and fluids, which differs from the carcinogenic Cr^6+^ that is soluble and uses phosphate channels to penetrate the cell membranes (Jobby et al., 2018). Cobalt in tissues is in the form of Co^2+^, which is mostly soluble and is an important cofactor for vitamin B12 (Cobalamin); it can penetrate cells, and interact with organelles and proteins (Czarnek et al., 2015).

**Figure 8:**
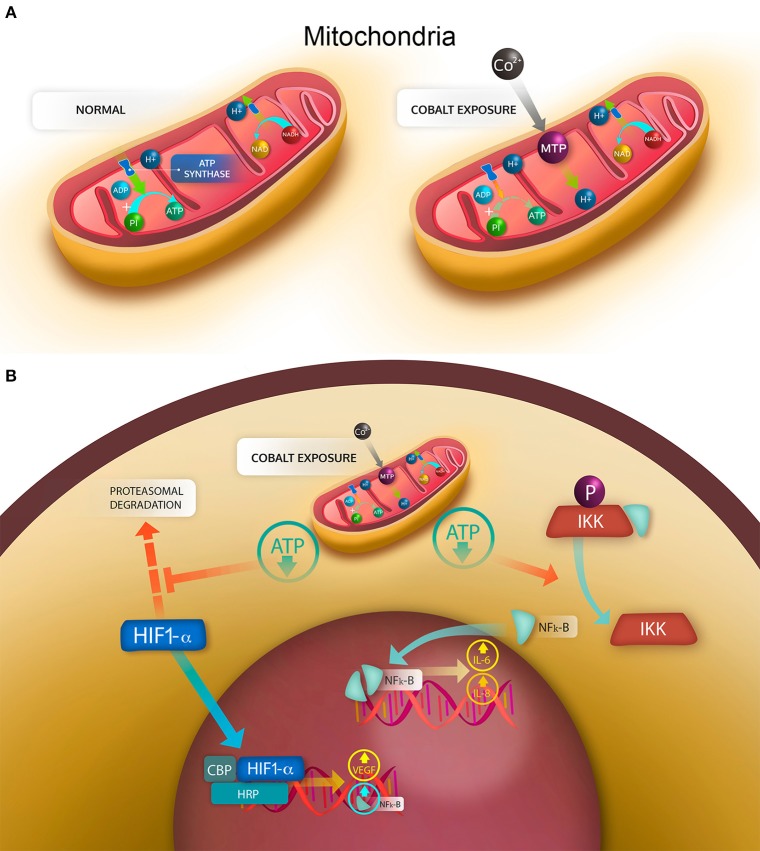
Mechanisms of cell damage induced by cobalt ions. **(A)** mitochondrial stress generated by the cobalt-induced opening of the mitochondrial transition pore (MTP). Under normal conditions in aerobic respiration, the H^+^ generated during the tricarboxylic acid cycle in the form of NADH and NAPH are transferred to the intermembrane space by the electron transport chain, generating an electrochemical gradient, in which a high concentration of H^+^ is found in the intermembrane space. By use of this electrochemical gradient, the ATP synthase can phosphorylate ADP into ATP. With the opening of MTP, the electrochemical gradient is lost, then the synthesis of ATP is decreased. **(B)** cellular effects of the mitochondrial stress. Under normal conditions, the hypoxia induced factor HIF-1α is degraded by the proteasome; similarly, phosphorylated IKK bound in the cytoplasm NF-κB. With a drop in ATP, the degradation of HIF-1α is decreased, allowing HIF-1α to translocate into the nucleus and bind CBP and HRP to enhance transcription of target genes, such as NF-κB and VEGF. The decrease in ATP also reverses the phosphorylation of IKK that in a non-phosphorylated state releases NF-κB, which dimerizes and translocates to the nucleus, binding DNA and enhancing the transcription of target genes, such as IL-6 and IL-8.

As previously explained, the elevated concentration of cobalt ions can have cytotoxic effects on several cell types (Granchi et al., 1998b; Huk et al., 2004; Catelas et al., 2005; Fleury et al., 2006; Posada et al., 2015), produce necrosis and apoptosis (Granchi et al., 1998a; Huk et al., 2004), and increase the production of ROS (Salloum et al., 2018; Chamaon et al., 2019). It has also been demonstrated that human macrophages exposed to Co^2+^ and Cr^3+^ ions have an increased expression of apoptotic proteins Bax-2, caspase 3 and caspase 8 and a decreased expression of the antiapoptotic protein BCL-2 (Petit et al., 2004). However, a recent study using primary synovial fibroblasts showed no induction of apoptosis even at high concentrations (1 mM), but evident metabolic stress and cytokine secretion even at low concentrations (0.1 mM) (Eltit et al., submitted). One limitation in the analyses is that most of the described results were obtained “*in vitro*” by using concentrations of cobalt of 0.5 mM, which is higher than the values observed “*in vivo*.” At lower and more “physiopathological” concentrations (1–10 μM), ATP synthesis has shown to be inhibited by cobalt in a dose-dependent manner. This happens not as the result of the inhibition of ATP synthase or the respiratory chain, but as consequence of the opening of the mitochondrial permeability transition pore (MPT), which allows the H^+^ to migrate from the intermembrane space to the mitochondria matrix, thus decreasing the electromotive force that activates the ATP synthase (Figure 8A) (Bragadin et al., 2007). As a consequence of this aerobic respiration failure, the production of ATP is decreased, and the anaerobic pathway for ATP generation is stimulated (Kurhaluk et al., 2019). This opening of MPT pore induced by cobalt also decreases the pool of NADH and NADPH in rat liver mitochondria, and may “leak” antioxidant proteins from the mitochondrial matrix (Battaglia et al., 2009). The compositions and functions of MPT in normally functioning mitochondria are unknown. But it is known that they constitute pore structures with a cut-off of 1.5 kDa that alter the permeability of the internal membrane, resulting in massive flux of protons to the internal chamber and the release of calcium and metabolites, with a consequent loss of function and swelling of the mitochondria (Baines and Gutiérrez-Aguilar, 2018). This mechanism has been recently proposed to be responsible for cell damage in the traumatic injury of central nervous system (Springer et al., 2018).

The presence of cobalt ions has also been associated with the stabilization of the α-subunit of hypoxia-inducible factor−1 (HIF-1α), and an increased expression of the HIF-1α regulated genes (Karovic et al., 2007). The activation of HIF-1α normally occurs in response to hypoxia conditions, activating a survival program, and promoting vascularization and hematopoiesis (Lukyanova and Kirova, 2015). The mechanisms by which the MPT activation would lead to HIF-1α stabilization in the presence of cobalt are still not fully understood, but as consequence of HIF-1α stabilization cells release cytokines that would promote neovascularization, inflammation and hematopoiesis (Seeber et al., 2011). Furthermore, recently HIF pathway has been demonstrated to be activated in a macrophage cell line after being exposure to cobalt alloy nanoparticles, and this activation was associated with an increase in the secretion of the cytokines transforming growth factor alpha (TNF-α), interleukin 1 beta (IL-1β) and vascular endothelial growth factor (VEGF) (Nyga et al., 2015). Taken together these data suggest that the inflammation and cell death observed in the ALTRs could be explained by the hypoxic stress induced by cobalt, which is capable of eliciting the synthesis and secretion of pro-inflammatory cytokines that recruit cells of the immune system. However, this hypothesis could not explain the presence of highly destructive ALTRs in MoP implants with minimum wear or corrosion and low cobalt ions in synovial fluid and blood. Neither could it explain the fact that some patients with elevated levels of cobalt and chromium due to wear or corrosion do not develop a necrotic reaction and do not present a lymphocytic infiltrate.

## A Mixed Model in the Etiology of ALTRs

In one of the early studies in hip pathology associated with MoM hip implants, Campbell et al. (2010) observed that most of the lesions could be explained by excessive wear of the articulating surfaces and subsequent presence of metals in the tissues, but some lesions were also present in low wear implants. The authors then proposed that those reactions in low wear implants could be due to a type IV (delayed) hypersensitivity reaction (Campbell et al., 2010). Six years later, in a thorough histological analysis of ALTRs, Ricciardi et al. (2016) classified the lesions into four groups: macrophage-dominated, mixed macrophage-lymphocytic with or without hypersensitivity features, and granulomatous pattern. They reported that ALTRs with a mixed infiltrate and hypersensitivity features were more frequent in non-MoM ALTRs and were associated with a shorter revision time (Ricciardi et al., 2016). Similarly, a study including ALTRs in MoM and MoP implants showed a shorter revision time, higher lymphocyte infiltration, higher necrosis and ALVAL score in the MoP group, which also had lower concentration of metals in serum (Eltit et al., 2017).

The observations explained above suggest that there are two main events that explain two types of possibly interrelated reactions. The first is the a physiopathological event of metal-induced inflammation of the hip joints, which affects a larger number of patients, mostly with MoM implants. The second event is associated with hypersensitivity reaction, which is less prevalent in MoM ALTRs, but correspond to most of ALTRs in MoP ALTRs and low wear MoM.

As described previously, cobalt ions can induce MPT, which is not a constitutional structure of the mitochondria, but is stabilized under pathologic conditions. The increased permeability of the mitochondrial internal membrane decreases the electrochemical gradient between the intermembrane space and the mitochondrial matrix. Subsequently the proton-motive force is reduced, and the generation of ATP is impaired (Figure 8). The reduced ATP provokes the activation of hypoxia response, characterized by the HIF-1α stabilization and the production of cytokines associated with this factor. There is an evidence that HIF-1α also increases the expression of NFκB, a transcription factor that elicits the synthesis and secretion of inflammatory cytokines (Mukandala et al., 2016) (Figure 8B). The cytokine secretion is also accompanied by an increase in the reactive oxygen species (ROS) in the cell, which is enhanced by the MPT stabilization, which allows antioxidant molecules such as glutathione to leave the mitochondria, reducing their protective capability. The intracellular increase in ROS can also trigger the secretion of proinflammatory cytokines and is known to activate endothelium and cells of the immune system (Mittal et al., 2014). The proinflammatory effects of the secreted cytokines has been demonstrated to induce migration in monocytes and activation of endothelial cells, thus generating inflammatory changes in the synovium (Eltit et al., submitted). This synovial inflammation has been observed in most of the patients with hip replacements and is especially frequent in patients with MoM hip implants, which also present with high levels of Co and Cr in synovial fluid and serum. It is thought to be the mechanism that trigger the macrophage-dominated lesions that are mostly observed in MoM ALTRs and are associated with high wear and elevated metal ions in plasma (Figures 7A–C).

Some patients develop a hypersensitivity reaction, initiated by the activation of T-cells, driving to a predominant Th1 reaction (Catelas et al., 2015). The specific antigen that triggers hypersensitivity in ALTRs has not been described, but the presence of tertiary lymphoid structures needs the specific activation of T-cell receptors that are highly specific. Because of this specificity and the traditionally accepted model for metal allergies (Saito et al., 2016), the presence of an hapten-carrier complex, in which metal elements combined with host proteins became a new antigen for T-cell receptors has been proposed for ALTRs. Similarly, chronic inflammation or necrosis can expose intracellular antigens that can activate T-cells triggering hypersensitivity reactions, as is observed in lupus (Kubota, 1998); thus the presence of hypersensitivity could also be secondary to the pre-existing chronic inflammation of the hip joints. The open question then is whether these two events (mitochondrial stress-induced inflammation and delayed hypersensitivity) are related or they are different pathologies that affect patients with different characteristics? It seems that cytotoxicity-associated ALTRs affect patients with elevated levels of Co and Cr while hypersensitivity ALTRs affect patients with genetic predisposition independent of the metal levels or cell damage. However, the evidence of hypersensitivity reactions as a consequence of chronic inflammation, together with the existence of ALTRs with mixed characteristics (metal particles and macrophage infiltrate, and also hypersensitivity-like features), supports the hypothesis of a combined pathogenesis. In this model, both etiological elements are related, and the development of hypersensitivity is a consequence of the pre-existing cell damage-driven chronic inflammation. In consequence, ALTRs should be considered as a single pathology with two pathogenic variances.

## Conclusion Remarks

It is accepted that CoCrMo over release from either tribocorrosion at the articular surfaces or fretting corrosion at the modular junctions of hip implants trigger adverse local tissue reactions. These mechanisms of degradation results in elevated metals ions in the periprosthetic tissues and fluids, which are the most likely species to induce cell damage. The generation of particles has an important role in metal ion release by re-passivating exposed surfaces, increasing the exposed surface, and inducing phagocytosis that expose the particles to an oxidative environment. Metal ions, mainly cobalt, induces mitochondrial stress, by opening transition pores in the mitochondrial internal membrane. The presence of pores in the membrane decrease the electrochemical potential reducing the ATP synthesis generating a hypoxia-like condition in the cells. Subsequently the transcription factors HIF-1α and NFκB trigger the synthesis and secretion of cytokines that elicit the inflammation in the periprosthetic tissues. The mechanisms of hypersensitivity are not clear, but activation of T-cell is highly specific, thus the presence of specific antigens that activates the lymphocyte response is needed. In summary, ALTRs are complex pathology, in which two major physiopathological events are involved: the mitochondrial stress induced by metal ions, and the development of hypersensitivity in a pre-existing chronic inflammation setting.

## Author Contributions

All authors contributed to reviewing the relevant literature and drafting the manuscript. Figures were obtained and processed by QW and FE.

### Conflict of Interest Statement

The authors declare that the research was conducted in the absence of any commercial or financial relationships that could be construed as a potential conflict of interest.
